# The effects of WeChat-based educational intervention in patients with ankylosing spondylitis: a randomized controlled trail

**DOI:** 10.1186/s13075-021-02453-7

**Published:** 2021-03-04

**Authors:** Yuqing Song, Xia Xie, Yanling Chen, Ying Wang, Hui Yang, Anliu Nie, Hong Chen

**Affiliations:** 1 West China School of Nursing/West China Hospital, Sichuan University, No. 37, Guoxuexiang, Wuhou District, Chengdu, 610041 Sichuan China; 2 Department of Nursing, Affiliated Hospital of North Sichuan Medical College, Nanchong, 637000 Sichuan China; 3 School of Nursing, North Sichuan Medical College, Nanchong, 637000 Sichuan China; 4 Department of Rheumatology and Immunology, West China Hospital, Sichuan University, No. 37, Guoxuexiang, Wuhou District, Chengdu, 610041 Sichuan China; 5 Nursing Department, The First Affiliated Hospital of Harbin Medical University, No.23 Youzheng Street, Nangang, Harbin, 150001 Heilongjiang China; 6 Emergency Department, The First Affiliated Hospital of Guangzhou Medical University, No. 151 Yanjiangxi Road, Guangzhou, Guangdong China

**Keywords:** Ankylosing spondylitis, Patient education, Telehealth, mHealth, Depression, Health-related quality of life

## Abstract

**Background:**

Ankylosing spondylitis (AS), as a common inflammatory rheumatic disease, often causes depression and impaired health-related quality of life (QoL). Although positive effects of patient education have been demonstrated, limited studies explored the benefits of education via mobile applications for AS patients. This study aimed to evaluate the effects of the WeChat-based educational intervention on depression, health-related QoL, and other clinical outcomes in AS patients.

**Methods:**

We conducted a single-blind randomized controlled trial from March to December 2017. Patients were recruited and randomized into the intervention group which received a 12-week WeChat-based educational intervention (consisting of four individual online educational sessions, online educational materials) or the control group receiving standard care. Data was collected at baseline and 12 weeks. Outcomes were measured by Beck Depression Inventory-II, the Medical Outcomes Study Short Form 36-item Health Survey (SF-36), Bath Ankylosing Spondylitis Functional Index (BASFI), Bath Ankylosing Spondylitis Patient Global Score (BAS-G), and visual analog scales.

**Results:**

A total of 118 patients with AS were included and analyzed. Measures at baseline were comparable between groups. After the intervention, the intervention group reported significant higher scores of all domains of SF-36 except for physical functioning and validity, compared with the control group. Additionally, patients in the intervention group had lower depressive symptoms than the control group. No significant difference in other outcomes was observed at 12 weeks.

**Conclusions:**

This study found that the 12-week educational intervention via WeChat had positive effects on reducing depressive symptoms and improving health-related QoL in Chinese patients with AS. We suggest that this intervention can be integrated into current routine care of AS patients.

**Trial registration:**

This study has been approved by the hospital’s ethics committee (ID: 20160364) in 2016 and registered at the Chinese Clinical Trail Registry (registry number: ChiCTR-IPR-16009293).

## Background

Ankylosing spondylitis (AS) is a common inflammatory rheumatic disease primarily affecting axial skeleton [1, 2]. AS starts in an early age and affects patients at their most productive age [3, 4]. The clinical features of AS mainly include inflammatory back pain, morning stiffness, functional impairment, and specific organ involvement. Besides, AS may have negative psychological consequences, such as depression and anxiety, and result in impaired health-related quality of life (QoL) [1, 4, 5]. Depressive symptoms are common among AS patients, with prevalence ranging from 11 to 64% [5, 6]. AS patients suffering from depressive symptoms might require intervention that is not recognized by rheumatologic doctors [6]. Moreover, evidence also indicates that AS patients have poorer health-related QoL compared with the general population, but similar to patients with other rheumatic conditions [1]. The European League Against Rheumatism (EULAR) recommends that the management of AS should aim at maximizing long-term health-related QoL through controlling symptoms and inflammation, preventing progressive structural damage and preserving function and social participation [2]. Thus, effective interventions to prompt AS patients’ QoL and depression are needed.

The best management for AS patients requires pharmacological and non-pharmacological treatment [2]. Patient education, as an effective, non-pharmacological treatment strategy for patients with AS, enables patients to manage their own conditions, enhances their ability to cope with disease, improves perceived health status, and maintains QoL [7]. Several previous studies explored the effects of educational interventions in patients with AS [8–13], but the effects of patient education on depression and quality of life are still unclear. Some studies used disease-specific measure to assess quality of life [8, 12], which cannot compare QoL among individuals with different health conditions [4]. The Medical Outcomes Study Short Form 36-item Health Survey (SF-36) is a generic instrument that can be used in the general population and various disease populations, and this scale enables us to compare health-related QoL in populations with different health conditions [4].

Educational interventions for AS patients are mainly provided through face-to-face mode, such as group education, peer-led education [8, 10, 11, 14]. Time constraints, physical limitation, and transportation cost are barriers for AS patients to attend these educational interventions [15, 16]. The use of mobile telehealth platforms is a new and innovative delivery strategy for patient education that may overcome the barriers of face-to-face strategy [17, 18]. In recent years, educational interventions delivered by mobile health (mHealth) approaches have grown rapidly with increased access to telecommunication devices, e.g., tablets and smartphones [15]. In China, WeChat, with more than 1 billion monthly active users, is the most widely and frequently used mobile application (app) [17, 18]. WeChat provides various services, such as instant messaging, free voice/video call, private or group chatting, browsing, and posting information [17, 18]. Patient education via WeChat is a useful tool to overcome current barriers in traditional face-to-face education [17, 18]. Moreover, Zhang et al. revealed that individualized health education through WeChat can play a central role in health education efforts in China because WeChat is well-integrated into society [18]. In recent years, some studies have found that interventions delivered by WeChat can improve health behaviors and health outcomes in patients with other chronic diseases [19–22]. Hu et al. [23] explored the transitional care delivered by WeChat in discharged patients with AS, but they educated patients only through group chatting. The individual educational intervention delivered via WeChat for patients with AS has not been well explored.

The purpose of this study was to develop and explore the benefits of a 12-week educational intervention delivered by WeChat on health-related quality of life measured by SF-36, depression measured by Beck Depression Inventory-II (BDI-II), and clinical outcomes among patients with AS in China.

## Methods

### Aim and study design

The aim of this study was to evaluate the effects of a 12-week patient education program delivered by WeChat on depression, quality of life, and clinical outcomes in Chinese patients with AS. This study was an assessor-blind, parallel-group, randomized controlled trial conducted between March and December 2017.

### Ethical considerations

This study was conducted in accordance with the Helsinki declaration. Ethical approval was obtained from West China Hospital Medical Ethics Committee in 2016(ID: 20160364). All participants were informed the content and procedure of this study and provided informed consent.

### Participants

Eligible patients were recruited from the Department of Rheumatology and Immunology at West China Hospital of Sichuan University, Chengdu, China. The inclusion criteria were as follows: fulfilling the modified New York classification criteria for AS [24], aged ≥ 14 years, able to understand/read Chinese, able to use the WeChat, and willing to participate in this study. We excluded patients who had severe psychological and cognitive impairment, and were participating in other studies. Patients with other rheumatic diseases were also excluded.

Patients were enrolled and randomly allocated into the intervention group or the control group using a randomization code generated by the Excel software. The assessors who collected data were blind to the allocation of the participants.

### Intervention group

Participants in the intervention group received the 12-week educational intervention delivered by WeChat and standard care. The educational intervention was mainly carried out by experienced research nurses who were well-trained the study protocol. The content of the educational intervention was developed on literature review, expert consultation, and a pilot study. The core content included: basic knowledge, exercise, medication, daily life management, psychological support, and self-assessment.

During the 12-week intervention, participants in the intervention group received four individual educational sessions and online educational information on WeChat platform. The four individual educational sessions were conducted through WeChat voice/video calls based on participants’ preference, and each session lasted 20–30 min depending on their willingness to communicate. At the first educational session, the research nurses assessed participants’ needs, problems, and barriers of managing their life. Then, participants were taught the knowledge and skills of managing their condition, including exercise, medication taking, daily life instruction, and psychological management strategies. The participants were also taught how to use validated instruments to assess their disease condition. During subsequent sessions, the research nurses taught participants strategies to manage their disease based on participants’ problems and health behaviors over the previous 2 or 4 weeks. In addition, the research nurses also sent online educational information including articles and videos to participants once a week. During the whole intervention phase, we encouraged participants to ask questions and share their experience at any time.

### Control group

The control group received standard care at hospital. The standard care consisted of basic health advice and brief guidance on medication and exercise. Participants in the control group could ask the researchers questions about disease via WeChat or mobile phone, but did not access to the educational program.

### Measures and data collection

All participants completed baseline assessments at the Department of Rheumatology and Immunology. Outcome measures, including quality of life, depression, overall well-being, physical function, morning stiffness, and pain, were collected at the baseline and after the 12-week educational intervention. Two well-trained research assistants who were blind to group allocation collected study data at baseline and after the 12-week educational intervention. If participants had difficulties in completing the questionnaires, the research assistants would help them read each item and record their responses.

### Participants characteristics

The baseline demographic data included age, gender, educational level, marital status, household income, medical insurance, and smoking status. Disease-specific data included disease duration (e.g., duration since diagnosis, symptom duration) and current medication.

### Quality of life

Health-related QoL was measured using the Medical Outcomes Study Short Form 36-item Health Survey (SF-36) [25, 26]. The SF-36 consists of 36 items and measures eight aspects of health status during the past 4 weeks. The eight domains of the SF-36 includes: physical functioning (PF), role physical (RP), bodily pain (BP), general health (GH), vitality (VT), social functioning (SF), role emotional (RE), and mental health (MH) [4, 25]. Each domain is scored ranging from 0 to 100, higher scores indicating better QoL [4]. This scale has been translated into different language versions and validated in patients with AS [4, 27]. In this study, Cronbach’s α of total score was 0.870, and Cronbach’s α of each domain ranged from 0.777 to 0.915.

### Depression

Depression was evaluated using the Chinese version of Beck Depression Inventory-II (BDI-II) [28]. The BDI-II is a validated self-reported questionnaire developed by Beck et al. [29]. It comprises 21 items and evaluates the severity of depressive symptoms during the past 2 weeks [30]. Each item score ranges from 0 to 3, and total score ranges from 0 to 63. Higher scores indicate severer depressive symptoms [28, 31]. In the current study, Cronbach’s α was 0.837.

### Disease specific measures

The Bath Ankylosing Spondylitis Patient Global Score (BAS-G) was used to assess the effect of AS on overall patients’ well-being over the last week and during the last 6 months [32]. The test-retest reliability of BAS-G was excellent, and construct validity and predictive validity was good [32]. The BAS-G score ranges from 0 to 10 and higher scores indicate worse patients’ global assessment. Physical function was measured by the Bath Ankylosing Spondylitis Functional Index (BASFI) [33]. The BASFI score ranges from 0 to 10, and higher scores indicate worse physical function [33]. Patients’ overall pain, back pain, nocturnal pain, and morning stiffness were collected using a visual analog scale, and final scores range from 0 (none) to 10 (severe).

### Statistical analysis

All data were analyzed using the Statistical Package for Social Sciences 22.0(SPSS Inc., Chicago, IL, USA). The intention-to-treat analysis (ITT) method was used to analyze data. Participants’ characteristics and outcome scores were summarized using means ± standard deviation (SD) or medians [interquartile ranges (IQR)] for continuous variables or frequencies (percentages) for categorical variables. Baseline characteristics and outcomes were compared using independent samples *t* test or Mann-Whitney *U* test from continuous variables and chi-squared test for categorical variables between the intervention and control group. *P* values < 0.05 were considered statistically significant.

## Results

### Participants’ flow through the trial

**Fig. 1:**
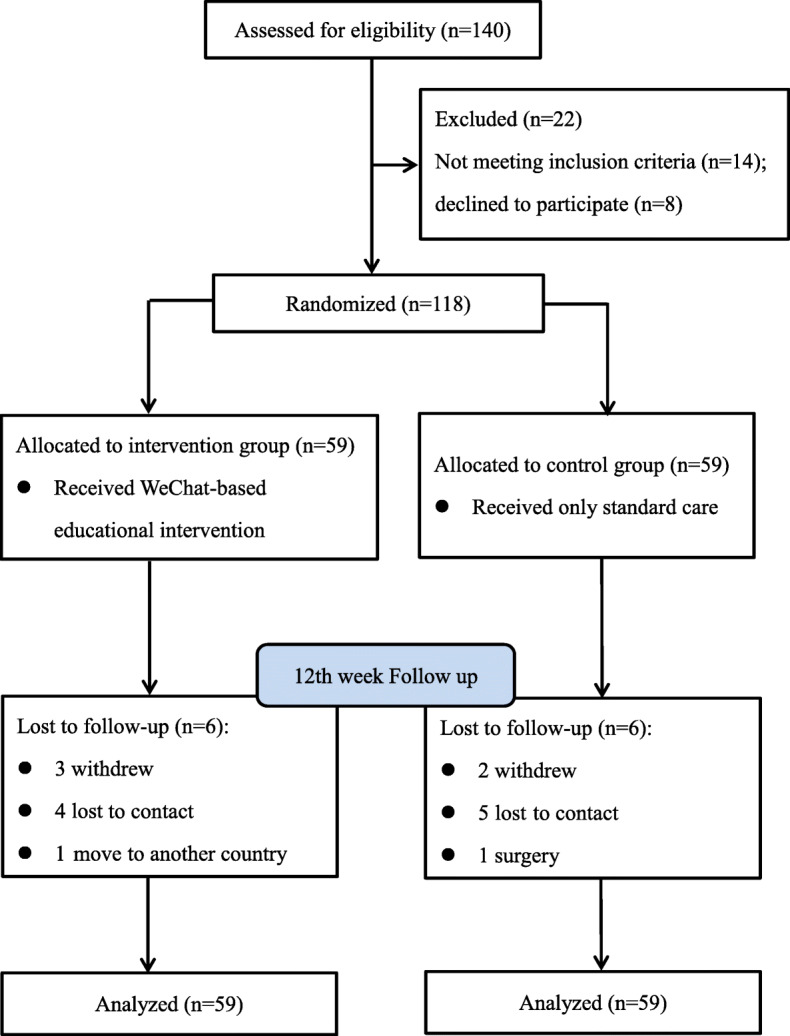
Flow diagram of the study

Figure 1 shows the flow diagram of the study. There were 140 potential participants assessed for eligibility and 22 excluded. We recruited 118 patients with AS and randomized them into the intervention group (*N* = 59) or control group (*N* = 59). Of 118 participants, 106 completed the 12-week educational intervention and post-test. We included 118 patients in statistical analysis since an ITT analysis was used.

### Participant baseline characteristics

The baseline characteristics of the participants are described in Table 1. The average age was 29.93 years, and the median years of symptom duration and duration since diagnosis were 5 and 3, respectively. Most of the participants were male, single/divorced, and had high educational level and no medical insurance. At the baseline, both groups were comparable since no statistically significant differences were observed in the variables between the two groups.

**Table 1:** Baseline characteristics of participants in the intervention and control group

Variable	All (*N* = 118) *N* (%)	Intervention group (*N* = 59) *N* (%)	Control group (*N* = 59) *N* (%)	*t*/*χ*^2^/*Z*	*P*
Age (yr, mean ± SD)	29.93 ± 8.23	30.80 ± 8.82	29.07 ± 7.58	1.142	0.256^a^
Gender				0.457	0.499^b^
Male	93 (78.8)	45 (76.3)	48 (81.4)		
Female	25 (21.2)	14 (23.7)	11 (18.6)		
Educational level				0.218	0.897^b^
Junior high school or below	30 (25.4)	15 (25.4)	15 (25.4)		
Senior high school	26 (22.0)	12 (20.3)	14 (23.7)		
College or above	62 (52.5)	32 (54.2)	30 (50.8)		
Marital status				0	1.000^b^
Single/divorced	62 (52.5)	31 (52.5)	31 (52.5)		
Married	56 (47.5)	28 (47.5)	28 (47.5)		
Monthly per capita income (¥, yuan)				1.720	0.633^b^
< 2200	34 (28.8)	16 (27.1)	18 (30.5)		
2200~3299	33 (28.0)	15 (25.4)	18 (30.5)		
3300~5499	23 (19.5)	11 (18.6)	12 (20.3)		
≥ 5500	28 (23.7)	17 (28.8)	11 (18.6)		
Medical insurance				0.160	0.689^b^
Self-pay	82 (69.5)	40 (67.8)	42 (71.2)		
Medical insurance	36 (30.5)	19 (32.2)	17 (28.8)		
Smoking status				1.502	0.472^b^
Current smoking	36 (30.5)	19 (32.2)	17 (28.8)		
Never smoking	70 (59.3)	36 (61.0)	34 (57.6)		
Quit smoking	12 (10.2)	4 (6.8)	8 (13.6)		
Medication				2.185	0.139^b^
DMARDs	64 (54.2)	28 (47.5)	36 (61.0)		
DMARDs+biologics	54 (45.8)	31 (52.5)	23 (39.0)		
Symptom duration (yr), M [IQR]	5.00 [6.00]	6.00 [7.00]	5.00 [7.00]	− 0.346	0.730^c^
Duration since diagnosis (yr), M [IQR]	3.00 [6.00]	3.00 [6.00]	3.00 [6.00]	− 0.577	0.564^c^
SF-36 subgroups					
Physical functioning	77.88 ± 18.67	77.54 ± 20.46	78.22 ± 16.86	− 0.196	0.845^a^
Role physical, M [IQR]	50.00 [100.00]	50.00 [100.00]	50.00 [100.00]	− 0.490	0.624^c^
Bodily pain	51.49 ± 20.31	53.97 ± 22.39	49.02 ± 17.84	1.328	0.187^a^
General health	49.08 ± 21.29	49.81 ± 21.93	48.34 ± 20.79	0.375	0.708^a^
Vitality	65.89 ± 18.95	67.63 ± 19.06	64.15 ± 18.85	0.996	0.321^a^
Social functioning	70.46 ± 22.68	69.33 ± 23.45	71.59 ± 22.03	− 0.539	0.591^a^
Role emotional	64.41 ± 43.52	66.67 ± 43.33	62.15 ± 43.97	0.562	0.575^a^
Mental health	67.29 ± 15.39	67.12 ± 15.97	67.46 ± 14.92	− 0.119	0.905^a^
BDI-II, M [IQR]	5.00 [11.25]	5.00 [10.00]	6.00 [12.00]	− 0.543	0.587^c^
BAS-G	3.36 ± 2.31	3.31 ± 2.37	3.41 ± 2.27	− 0.234	0.815^a^
BASFI, M [IQR]	0.60 [1.70]	0.60 [1.90]	0.60 [1.50]	− 0.586	0.558^c^
Overall pain	3.24 ± 2.35	2.97 ± 2.33	3.51 ± 2.36	− 1.267	0.208^a^
Back pain	2.96 ± 2.33	2.76 ± 2.30	3.16 ± 2.36	− 0.929	0.355^a^
Nocturnal pain	3.02 ± 2.48	3.00 ± 2.33	3.05 ± 2.64	− 0.103	0.918^a^
Morning stiffness	2.45 ± 2.11	2.35 ± 1.93	2.56 ± 2.29	− 0.545	0.587^a^

*SD* standard deviation, *yr* year, *DMARDs* disease-modifying anti-rheumatic drugs, *M* median, *IQR* interquartile range, *BDI-II* Beck Depression Inventory-II, *BAS-G* Bath Ankylosing Spondylitis Patient Global Score, *BASFI* Bath Ankylosing Spondylitis Functional Index

^a^Independent samples *t* test

^b^Chi-square test

^c^Mann-Whitney *U* test

### Outcomes

As shown in Table 2, role physical, bodily pain, general health, social functioning, role emotional, and mental health scores of SF-36 in the intervention group were significantly higher than that of the control group after the intervention (all *P* < 0.05). BDI-II score was lower in the intervention than those in the control group (*P* < 0.05). However, there were no statistically significant differences in other outcomes between the two groups (*P* > 0.05).

**Table 2:** Comparison of outcomes between the intervention and control group

Variable	Intervention group (*N* = 59) Mean ± SD	Control group (*N* = 59) Mean ± SD	*t*/*Z*	*P*
SF-36 subgroups				
Physical functioning	83.05 ± 16.24	79.41 ± 15.68	1.240	0.217^a^
Role physical, M [IQR]	100.00 [50.00]	50.00 [100.00]	− 2.429	**0.015**^b^
Bodily pain	65.75 ± 16.85	55.29 ± 17.17	3.340	**0.001**^a^
General health	59.05 ± 17.73	49.95 ± 20.71	2.565	**0.012**^a^
Vitality	70.25 ± 15.04	65.34 ± 18.19	1.600	0.112^a^
Social functioning	83.31 ± 18.30	73.54 ± 18.08	2.917	**0.004**^a^
Role emotional	81.36 ± 34.61	61.02 ± 43.84	2.797	**0.006**^a^
Mental health	72.88 ± 13.96	65.97 ± 15.64	2.534	**0.013**^a^
BDI-II, M [IQR]	3.00 [5.00]	5.00 [13.00]	− 1.980	**0.048**^b^
BASFI, M [IQR]	1.00 [1.40]	1.40 [1.60]	− 1.764	0.078^b^
BAS-G	3.28 ± 2.10	3.68 ± 1.94	− 1.060	0.291^a^
Overall pain	3.05 ± 1.87	3.25 ± 1.92	− 0.583	0.561^a^
Back pain	3.19 ± 2.16	2.93 ± 2.03	0.658	0.512^a^
Nocturnal pain	2.83 ± 2.10	2.86 ± 2.18	− 0.086	0.932^a^
Morning stiffness	2.42 ± 1.93	2.64 ± 1.64	− 0.672	0.503^a^

*SD* standard deviation, *M* median, *IQR* interquartile range, *SF-36* the Short Form 36-item Health Survey, *BDI-II* Beck Depression Inventory-II, *BASFI* Bath Ankylosing Spondylitis Functional Index, *BAS-G* Bath Ankylosing Spondylitis Patient Global Score

^a^Independent samples *t* test

^b^Mann-Whitney *U* test

## Discussion

We developed and evaluated the effects of the 12-week educational intervention delivered by WeChat among AS patients in China. The results demonstrated that the 12-week educational intervention was able to improve health-related QoL and reduce depressive symptoms, but no significant effects on physical function, overall well-being, pain, and morning stiffness. Our finds suggested that WeChat was a feasible and effective method to deliver health service for Chinese patients with AS in clinical setting.

Patient education via WeChat is more convenient, timely, and cost-effective, because WeChat has various interactive functions and numerous users [18]. During four individual educational sessions, WeChat supported interactive and effective communication between researchers and participants. Meanwhile, we were able to send health information to participants according to their preference, e.g., via text, pictures, article, voice, and video, which could meet their educational needs. This intervention educated participants knowledge and skills of disease management, helped them understand disease well, and might contribute to better health outcomes.

We found that the WeChat-based educational intervention had a positive effect on reducing depressive symptoms in patients with AS, which was in line with several studies among AS patients [14, 23]. Hu et al. [23] revealed that transitional care delivered by WeChat could decrease depression and anxiety in discharged patients with AS. Similarly, WeChat-based interventions also have significant effects on reducing depression in people living with HIV [34], cancer patients [21], and pregnant women [35]. The education delivered by social media platform, WeChat, can optimize communication between patients and health care professionals and teach patients psychological management strategies [18, 23], which might improve psychological outcomes. Additionally, the nurse-led educational intervention may cause positive psychological outcomes. Kaya et al. [10] found that a peer-led education did not alert depression scores among AS patients. EULAR’s recommendations support that nurses play a crucial role in addressing patients’ psychological issues to reduce their symptoms of depression and anxiety [36]. Dures et al. [37] found that around three-quarters of patients with inflammatory arthritis prefer for psychological support delivered by rheumatology nurses. In the current study, the educational intervention was led by nurses who well understood patients’ needs and psychological concerns. The research nurses helped participants express their psychological concern and taught them how to deal with psychological distress. Thus, this educational intervention could reduce participants’ depressive symptoms.

Some studies revealed that educational programs are effective for AS patients to improve health-related QoL [8, 11, 13], but others found reverse results [9, 10, 12]. Our study found that all domain scores of quality of life, except for physical function and vitality, were significantly higher in the intervention group compared with the control group after 12-week intervention, which means the education via WeChat can effectively improve QoL. This result was similar to previous WeChat-based interventions for other chronic conditions [22, 38, 39]. Wang et al. [38] randomized 400 patients after hip replacement surgery into control group receiving routine nursing care or intervention group receiving continuous intervention via the WeChat-based orthopedic care platform and found that the 6-month continuous intervention via WeChat elevates patients’ quality of life. Sui et al. [39] revealed that WeChat app-based education and rehabilitation program is an effective way to improve quality of life in non-small cell lung cancer patients after undergoing surgical resection. Psychological distress, especially depression, has significant impacts on QoL [1]. This intervention reduced the symptoms of depression, which may contribute to better QoL. Meanwhile, this intervention taught patients knowledge and skills of managing their disease, such as regular treatment, adherence to exercise, and health behaviors. Regular treatment and physical exercise are beneficial to the mental and physical aspects of QoL in AS patients [1]. Thus, this intervention could help patients cope with their health condition and improve physical and mental health.

This intervention did not have significant effects on physical functioning and validity domains of QoL. Physical functioning of QoL reflects limitations in physical activities because of health problems and is influenced by functional status [25, 27]. Validity reflects perceived energy and fatigue and is associated with patients’ disease activity, physical function, and morning stiffness [27, 40]. In the current study, the educational intervention via WeChat did not affect patients’ physical function, pain, and morning stiffness. Thus, the intervention may not significantly improve physical functioning and validity domains of QoL.

We did not detect the short-term effects of the intervention on physical function, overall well-being, pain, and morning stiffness in AS patients. Similarly, Kaya et al. [10] and O’Dwyer et al. [12] found that education programs targeting at AS patient have no significant effects on health status outcomes (e.g., pain, BAS-G, disease activity) in a short-term period. Zhou et al. [22] revealed that the WeChat-based nursing program does not significant improve pain, fatigue, and sleep disturbance of postoperative women with breast cancer in 6 months of surgery. Patient education may have positive effects on patients’ disease control in a long time [41]. In the current study, we only explored the short-term effects and found no significant positive results on physical function, overall well-being, pain, and morning stiffness. The long-term effects of the intervention remain unknown. Thus, we suggest that future studies should explore the long-term effects of educational intervention on health status in AS patients.

### Limitations

The strength of this study was that we used an assessor-bind randomized controlled trial study design to explore effects of the intervention. However, this study also has several limitations. First, this study was limited by a small sample size and all participants from a tertiary hospital due to limited resources, which may affect the generalizability of our findings. Second, an important limitation is the short follow-up period of 12 weeks. Thus, we did not ensure if the effect of the intervention sustains after the end of the intervention. Third, our study only compared the effects of educational intervention delivered by WeChat with standard care, but it is also important to compare the benefits of different educational methods, such as group education, education via telephone call, and web-based education. Although the socioeconomic benefits of the education program are important, we did not conduct a full cost-benefit analysis because of the limited resource. Finally, we did not collect laboratory data, including erythrocyte sedimentation rate (ESR), C-reactive protein (CRP), Platelet, and HLA-B27, because this study did not have adequate financial support.

### Implications

Our findings supported that WeChat was a feasible and effective approach to deliver patient education in China [42, 43]. Healthcare providers can monitor patients remotely and enable immediate care delivery [44]. Moreover, AS patients’ characteristic and the wide use of WeChat make it more feasible to conduct WeChat-based education. The WeChat-based educational intervention can be improved by seeking feedback and opinions from participants in this study. We suggest this intervention should be integrated into clinical care. In the current study, researchers contacted with each patient and sent educational information to patients one by one, which may add significant time and work to the research. WeChat Mini Programs and WeChat Official Account are lightweight micro-apps on WeChat and support multiple functions [45, 46]. We suggest that WeChat mini programs and WeChat Official Account can be used to improve the WeChat-based intervention in the future.

## Conclusion

This study found that the 12-week educational intervention via WeChat had positive effects on reducing depressive symptoms and improving health-related QoL in Chinese patients with AS. This intervention can be integrated into current routine care of AS patients. Further multicenter studies should explore the long-term effects of education delivered by WeChat.

## Data Availability

Data used in the current study are available from the corresponding author on reasonable request.

## Abbreviations

QoL: Quality of life
AS: Ankylosing spondylitis
mHealth: Mobile health
app: Application
BDI-II: Beck Depression Inventory-II
SF-36: The Medical Outcomes Study Short Form 36-item Health Survey
BAS-G: Bath Ankylosing Spondylitis Patient Global Score
BASFI: Bath Ankylosing Spondylitis Functional Index (BASFI)
PF: Physical functioning
RP: Role physical
BP: Bodily pain
GH: General health
VT: Vitality
SF: Social functioning
RE: Role emotional
MH: Mental health
DMARDs: Disease-modifying anti-rheumatic drugs
SD: Standard deviation
M: Median
IQR: Interquartile ranges
