# Correction to: The effects of WeChat-based educational intervention in patients with ankylosing spondylitis: a randomized controlled trial

**DOI:** 10.1186/s13075-021-02523-w

**Published:** 2021-05-25

**Authors:** Yuqing Song, Xia Xie, Yanling Chen, Ying Wang, Hui Yang, Anliu Nie, Hong Chen

**Affiliations:** 1 West China School of Nursing/West China Hospital, Sichuan University, No. 37, Guoxuexiang, Wuhou District, Chengdu, 610041 Sichuan China; 2 Department of Nursing, Affiliated Hospital of North Sichuan Medical College, Nanchong, 637000 Sichuan China; 3 School of Nursing, North Sichuan Medical College, Nanchong, 637000 Sichuan China; 4 Department of Rheumatology and Immunology, West China Hospital, Sichuan University, No. 37, Guoxuexiang, Wuhou District, Chengdu, 610041 Sichuan China; 5 Nursing Department, The First Affiliated Hospital of Harbin Medical University, No.23 Youzheng Street, Nangang, Harbin, 150001 Heilongjiang China; 6 Emergency Department, The First Affiliated Hospital of Guangzhou Medical University, No. 151 Yanjiangxi Road, Guangzhou, Guangdong China

**Correction to: Arthritis Res Ther 23, 72 (2021)**

**https://doi.org/10.1186/s13075-021-02453-7**

Following publication of the original article [1], a typo was found in the Article Title and Trial Registration section of the Abstract.

The word “trail” should be corrected to “trial”.

The original article has been corrected.
